# Applications of Cu^2+^-Loaded Silica Nanoparticles to Photothermal Therapy and Tumor-Specific Fluorescence Imaging

**DOI:** 10.3390/jfb15040081

**Published:** 2024-03-25

**Authors:** Ji-Ho Park, Yejin Sung, SeongHoon Jo, Seung Ho Lee, Ju Hee Ryu, In-Cheol Sun, Cheol-Hee Ahn

**Affiliations:** 1 NanoBio Materials Laboratory, Department of Materials Science and Engineering, College of Engineering, Seoul National University, Seoul 08826, Republic of Korea; jikl1028@kist.re.kr (J.-H.P.);; 2 Medicinal Materials Research Center, Biomedical Research Division, Korea Institute of Science and Technology, Seoul 02792, Republic of Korea; dpwls319@kist.re.kr (Y.S.);; 3 Biomaterials Research Center, Biomedical Research Division, Korea Institute of Science and Technology, Seoul 02792, Republic of Korea

**Keywords:** photothermal therapy, tumor-specific imaging, copper ions, silica nanoparticles

## Abstract

Copper-based nanomaterials have been employed as therapeutic agents for cancer therapy and diagnosis. Nevertheless, persistent challenges, such as cellular toxicity, non-uniform sizes, and low photothermal efficiency, often constrain their applications. In this study, we present Cu^2+^-loaded silica nanoparticles fabricated through the chelation of Cu^2+^ ions by silanol groups. The integration of Cu^2+^ ions into uniformly sized silica nanoparticles imparts a photothermal therapy effect. Additionally, the amine functionalization of the silica coating facilitates the chemical conjugation of tumor-specific fluorescence probes. These probes are strategically designed to remain in an ‘off’ state through the Förster resonance energy transfer mechanism until exposed to cysteine enzymes in cancer cells, inducing the recovery of their fluorescence. Consequently, our Cu^2+^-loaded silica nanoparticles demonstrate an efficient photothermal therapy effect and selectively enable cancer imaging.

## 1. Introduction

Cancer remains a formidable global health challenge, demanding innovative approaches for both diagnosis and treatment. In the pursuit of efficient cancer treatment, diverse therapeutic modalities undergo continuous development. Among various preclinical treatment methods, photothermal therapy stands out as noteworthy. Photothermal therapy (PTT) aims to eradicate cancer cells by harnessing the heat energy generated by photothermal agents upon exposure to light. In order to efficiently annihilate cancer cells while minimizing damage to normal cells, PTT requires photothermal agents that possess the intrinsic capability to generate heat upon exposure to light. The development and application of such agents, exhibiting proficient photothermal properties, are pivotal determinants for the success of photothermal therapy.

Nanomaterials have emerged as promising candidates for photothermal therapeutic agents owing to their distinctive physicochemical properties and multifunctionality [1,2]. Notably, noble metal nanoparticles are frequently employed in PTT due to their ability to generate heat through the formation of a dielectric field when exposed to near-infrared (NIR) light [3,4,5]. Furthermore, the functionalities of noble metal nanoparticles can be tailored based on their size, shape, and materials for specific purposes. For instance, the size of nanoparticles affects the enhanced permeability and retention (EPR) as well as the fluorescence quenching effect, thereby enhancing the efficiency of drug delivery and cancer imaging, respectively [6,7]. Among noble metals, copper has been utilized as a PTT agent in various forms such as copper oxide (CuO) and copper sulfide (CuS) [8,9]. It converts NIR light into heat through the mechanism of the d-d transition of copper ions [10].

However, current copper-based materials have limitations in their applications as photothermal agents. CuO exhibits notably higher cytotoxicity compared to other metal oxides [11,12]. Although photothermal agents composed of CuS are recognized for their enhanced biocompatibility due to their biodegradability [13,14], they manifest low photothermal efficiency, necessitating intense laser power exceeding 14 W/cm^2^ to achieve a sufficient photothermal effect [15]. Such elevated laser power has the potential to inflict damage even on normal cells [16]. Moreover, current methods for the fabrication of copper-based nanoparticles commonly utilize toxic surfactants, rendering them unsuitable for biomedical applications without additional surface modification [17]. Consequently, there is an imperative need for the development of more effective copper-based photothermal agents.

In this study, we engineered silica nanoparticles loaded with Cu^2+^ ions for the photothermal therapy of cancer cells. Silica coating is commonly used for the surface modification of nanoparticles, as it enhances their biocompatibility [18]. Silica nanoparticles, therefore, serve as an ideal platform for the introduction of toxic copper ions. Previous research has demonstrated that silica coating can improve the photostability of copper-based nanoparticles and contribute to the production of more stable nanoparticles [15]. Building upon this methodology, we incorporated Cu^2+^ ions during the synthetic process of silica nanoparticles. The introduction of Cu^2+^ ions into silica nanoparticles during synthesis was significant, given that silica coating effectively mitigates the toxicity associated with copper ions. Consequently, Cu^2+^-loaded silica nanoparticles exhibited superior biocompatibility and an extended circulation time within the biological system. Notably, Cu^2+^ ions efficiently generated heat under continuous-wave NIR laser irradiation and quenched the fluorescence of fluorophores in close proximity to the silica nanoparticles, while they did not compromise biocompatibility.

Furthermore, the surface of the Cu^2+^-loaded silica nanoparticles was functionalized with amine groups to enable the chemical attachment of tumor-specific NIR probes (NIR-Cu-SiNPs) designed for selective tumor imaging. These probes comprised an NIR dye (Cy5.5) and a short peptide sequence, cleavable by cysteine protease enzymes such as cathepsin B and papain. The fluorescence of the NIR dye was quenched by Cu^2+^ ions through the Förster resonance energy transfer (FRET) mechanism [19]. Because cysteine protease enzymes were known to be overexpressed within cancer cells [20,21,22,23], the tumor-specific NIR probes selectively recovered their fluorescence only in the tumor tissue. Moreover, the utilization of NIR fluorescence for in vivo bioimaging offers advantages such as deep tissue penetration, minimal interference from other biomaterials, and reduced photo-damage [24]. Finally, we demonstrated the selective NIR tumor imaging ability and effective photothermal therapeutic effect of Cu^2+^-loaded silica nanoparticles.

The integrated features of Cu^2+^-loaded silica nanoparticles designate them as promising candidates for multifaceted cancer treatment and diagnosis. Their distinctive attributes, such as the photothermal effect and selective quenching/recovery of fluorescence, present a versatile platform for cancer imaging and therapy. This multifunctionality of these nanoparticles offers a dual benefit for therapeutic and diagnostic applications. This study serves as a pivotal contribution by not only addressing the prevailing challenges associated with copper-based nanomaterials but also demonstrating the sophisticated design that enables simultaneous imaging and treatment of cancer. 

## 2. Materials and Methods

### 2.1. Materials

Tetraethyl orthosilicate (≥99.0%), 3-aminopropyl triethoxysilane (97%), ammonium hydroxide solution (28.0~30.0%), N-(3-dimethylaminopropyl)-N′-ethylcarbodiimide hydrochloride (commercial grade), N-hydroxysulfosuccinimde sodium salt (sulfo-NHS; ≥98%), copper(II) chloride (99.995%), and Papain (10 units/mg protein) were obtained from Sigma Aldrich (St. Louis, MO, USA). Ethanol and Cy5.5-NHS fluorescent dye were obtained from Duksan (Ansan, Republic of Korea) and Bioacts (Incheon, Republic of Korea), respectively. RPMI-1640 media, fetal bovine serum, penicillin, and streptomycin were purchased from WELGENE Inc. (Daegu, Republic of Korea). DMEM media was commercially purchased from Genedepot (Barker, TX, USA). Hoechst 33342 was purchased from Invitrogen (Thermofisher Scientific; Waltham, MA, USA). The 4T1 (murine breast adenocarcinoma) and HDF (human dermal fibroblast) cells were purchased from ATCC (American Type Culture Collection; Manassas, VA, USA). TEM grid (Carbon Film 200 Mesh copper) was purchased from Electron Microscopy Sciences (Atlanta, GA, USA). Six-week-old female Balb/c nude mice were purchased from OrientBio, Inc. (Seongnam, Republic of Korea). Customized enzyme-cleavable peptides with NIR dye (Cy5.5-Gly-Phe-Leu-Gly-Gly-Lys-Gly-Gly-NHS) were ordered from Peptron (Daejeon, Republic of Korea). All chemicals were used without any purification. 

### 2.2. Instruments

The hydrodynamic size and optical properties were measured in Zetasizer Nano ZS (Malvern Instruments, Malvern, UK) and Cary 300 UV-Vis (Agilent, Santa Clara, CA, USA), respectively. The photothermal effect was induced with an 808-nm CW laser (CNI laser, Changchun, China). A confocal laser scanning microscope (Leica TCS SP8, Leica Microsystems GmbH, Wetzlar, Germany) and a microplate reader (VERSAmaxTM, Molecular Devices Corp., Sunnyvale, CA, USA) were used to observe the in vitro cell imaging test. The fluorescence recovery by enzymatic probe cleavage was observed by using the iBrightTM Imaging System (Invitrogen by Thermo Fisher Scientific, MA, USA). The fluorescence and temperature were measured in IVIS Lumina (Caliper LifeSciences, Waltham, MA, USA) and an infrared thermal imaging camera (FLIR, Wilsonville, OR, USA), respectively. The centrifuge was performed using Avanti J-E Series (Beckman Coulter, IN, USA). 

### 2.3. Synthesis of Cu^2+^-Loaded Silica Nanoparticles

The chemical synthesis was conducted at room temperature if not otherwise specified. Tetraethyl orthosilicate (3.2 mL) and ammonium hydroxide solution (3.2 mL, 30%) were mixed in 40 mL of ethanol under sonication for 2 h. Then, the colloid was centrifuged at 14,000 rpm for 20 min, washed with ethanol, and re-dispered with sonication. This process was repeated three times. After that, 5.44 g of copper(II) chloride was added to the silica nanoparticle colloid (40 mL) under stirring (8000 rpm) overnight. When the color of the mixture turned green, the reaction mixture was centrifuged at 14,000 rpm for 20 min and re-dispersed in 40 mL of co-solvent (ethanol: ammonium hydroxide solution = 1:1) to remove excessive copper(II) chloride. If the color of the suspension changed from green to blue, the colloid was washed with ethanol three times with centrifugation (14,000 rpm, 20 min). Given the density of silica and the measured size of synthesized silica nanoparticles, it was calculated that the concentration of nanoparticles was 7.34 × 10^11^ particles/mL.

### 2.4. Synthesis of Tumor-Specific NIR Probes Using Cu^2+^-Loaded Silica Nanoparticles (NIR-Cu-SiNPs)

The surface of Cu^2+^-loaded silica nanoparticles was functionalized with amine groups to introduce the customized enzyme-cleavable peptides with NIR dye. Because NHS esters selectively react with primary amine groups, the enzyme-activable probes with NHS were chemically attached to the amine groups on the surface of Cu^2+^-loaded silica nanoparticles forming a stable amide bond. First, 16 mL of Cu^2+^-loaded silica nanoparticles were mixed with tetraethyl orthosilicate (0.373 mL) and ammonium hydroxide solution (0.373 mL) under sonication for 2 h. Then, 3-aminopropyl trimethoxysilane (3.68 mL) was added to the reaction mixture, followed by the addition of ammonium hydroxide solution (3.68 mL). After sonication for 2 h, amine-functionalized silica nanoparticles were centrifuged at 14,000 rpm for 20 min and washed with ethanol three times. 

Then, the customized enzyme-cleavable probes were chemically conjugated to the Cu^2+^-loaded silica nanoparticles to form the tumor-specific NIR probes using Cu^2+^-loaded silica nanoparticles (NIR-Cu-SiNPs). 3 mL of Cu^2+^-loaded silica nanoparticles were centrifuged at 14,000 rpm for 10 min and the solvent was changed to water. Then 300 μL of tumor-specific NIR probe stock solution (80 μg/mL) was added under stirring at 45 °C for 2 h. Then, the reaction mixture was centrifuged at 14,000 rpm for 10 min and washed with water two times to remove unreacted NIR probes. The concentration of Cy5.5 in the supernatant was analyzed with UV-vis spectroscopy to calculate the amount of tumor-specific NIR probes introduced on the surface of nanoparticles.

### 2.5. Photothermal Effect and Fluorescence Recovery of NIR-Cu-SiNPs

NIR-Cu-SiNPs with various concentrations (16, 32, and 64 mg Cu^2+^/mL) were dispersed in phosphate-buffered saline (PBS) after centrifugation and irradiated by an 808-nm laser (1.5 W/cm^2^) for 10 min. Then, the temperature change by photothermal heat generation was monitored with an infrared thermal imaging camera. Also, the enzyme-specific fluorescence recovery of NIR-Cu-SiNPs was evaluated. Then, 100 μL of NIR-Cu-SiNPs (1.5 mg Cu^2+^/mL) was placed in the 96-well cell culture plate and 0.75 units of papain were added at 37 °C. The enzymatic cleavage reaction was observed within 0.5 h after the addition of papain in the iBrightTM Imaging System for the calculation of fluorescence recovery.

### 2.6. Cytotoxicity and In Vitro Hyperthermia Test

The cell viability of NIR-Cu-SiNPs was evaluated by cell counting kit-8 (CCK-8) assays and crystal violet solution staining. First, 8 × 10^3^ 4T1 cancer cells were seeded in the 96-well cell culture plates and were cultured at 37 °C, 5% CO_2_ in RPMI-1640 medium containing fetal bovine serum (10%, *v*/*v*) and antibiotic/antimycotic (1%, *v*/*v*). After 24 h, cells were treated with NIR-Cu-SiNPs varying their concentrations for 4 h. Then, the cells were washed with PBS three times and incubated for 2 h with a culture medium containing 10% CCK-8 solution according to the manufacturer’s instructions. The cell viability was counted at 450 nm using a microplate reader. Also, live cell viability was evaluated with the same procedure except that culture media contained a crystal violet solution (1%, *v*/*v*), instead of the CCK-8 solution. The 4T1 cancer cells stained with crystal violet were imaged with a microscope. For the hyperthermia test, cells were treated with 32 mg Cu^2+^/mL for 4 h and washed with PBS before they were irradiated with an 808-nm laser (1.5 W/cm^2^) for 10 min. Then, cells were incubated for another 2 h after CCK-8 or crystal violet solution was applied. Then, their cell viability was recorded and a one-way or two-way analysis of variance (ANOVA) was performed using Microsoft^®^ Excel^®^ software (ver. 16.0.10407.20032).

### 2.7. In Vitro Fluorescence Cell Imaging

The cellular uptake and fluorescence recovery of NIR-Cu-SiNPs were evaluated with the confocal microscope. 1 × 10^5^ 4T1 cancer cells or 3 × 10^5^ human dermal fibroblasts were seeded on glass-bottomed confocal dishes (Cat NO. 200350, SPL Life Science, Daejeon, Republic of Korea) and incubated at 37 °C, 5% CO_2_ in RPMI-1640 medium containing fetal bovine serum (10%, *v*/*v*) and antibiotic/antimycotic (1%, *v*/*v*) for 24 h. The cell culture media was removed with suction and the 100 μL of NIR-Cu-SiNPs (2.9 mg Cu^2+^/mL) was treated in the cells for 0, 0.5, 1, 2, and 4 h. Then, the cells were washed with PBS and treated with a 1% solution of Hoechst 33342 for 10 min to stain the nucleus of the cells. Then, the cells were washed with PBS three times and the fluorescence was analyzed by a confocal microscope.

### 2.8. In Vivo Photothermal Therapy

Animal experiments were conducted in compliance with the relevant laws and institutional guidelines of the Institutional Animal Care and Use Committee at the Korea Institute of Science and Technology under protocol 2022-04-5052 (approval date: 8 June 2023). 4T1-bearing balb/c nude mice (6 weeks old, OrientBio, Inc., Seongnam, Republic of Korea) were prepared by the inoculation of 8 × 10^5^ cells on the breast of twenty mice. When the tumor size reached approximately 100 mm^3^ after 10~12 days, 30 μL of NIR-Cu-SiNPs (64 mg Cu^2+^/mL) in PBS was intratumorally injected. For photothermal therapy, an 808 nm laser (1.5 W/cm^2^) was irradiated on the tumoral site for 5 min. At 24 h after the irradiation, the mice were sacrificed and the tumor tissues were extracted. The tumors were fixed in PBS containing 4% paraformaldehyde. The samples were embedded in paraffin and sectioned at a thickness of 10 μm. They were stained with hematoxylin and eosin for the measurement of structural damages by photothermal therapy using an optical microscope.

### 2.9. In Vivo Fluorescence Imaging

4T1-bearing animal models were also prepared to image the tumor-specific fluorescence recovery. Likewise, 4T1 cancer cells with the number of 8 × 10^5^ cells were inoculated into twenty 6-week-old balb/c nude mice (OrientBio, Inc., Seongnam, Republic of Korea). After 10~12 days, the tumor size reached approximately 100 mm^3^ and 30 μL of NIR-Cu-SiNPs (64 mg Cu^2+^/mL) in PBS was injected into the tumor and its surrounding tissues. Then, the fluorescence of the whole body of the mice was monitored with an IVIS instrument. 

## 3. Results & Discussion

**Scheme 1:**
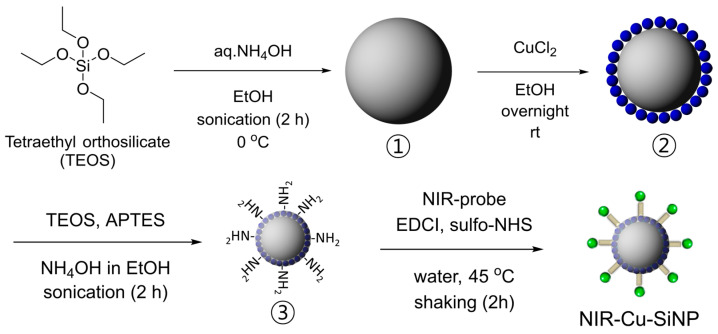
A schematic illustration depicting the synthesis of NIR-Cu-SiNPs (APTES: 3-aminopropyl triethoxysilane (97%), EDCI: N-(3-dimethylaminopropyl)-N’-ethylcarbodiimide hydrochloride, sulfo-NHS: N-hydroxysulfosuccinimde sodium salt, ①: silica nanoparticle, ②: Cu^2+^-loaded silica nanoparticle, ③: Cu^2+^-loaded silica nanoparticle with amine functionalization).

**Figure 1:**
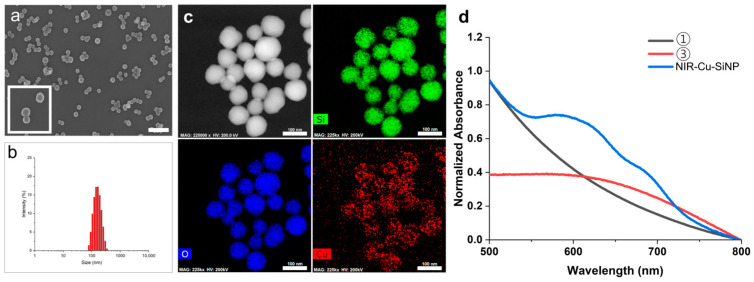
Characterization of NIR-Cu-SiNP. (**a**) SEM images revealed the spherical morphology of NIR-Cu-SiNPs (scale bar = 500 nm). (**b**) DLS measurements demonstrated a diameter of 163.7 ± 1.04 nm for NIR-Cu-SiNPs. (**c**) TEM and EDS mapping illustrated the presence of O, Si, and Cu elements within the nanoparticles (scale bar = 100 nm). (**d**) UV-Vis spectra, represented by black, red, and blue curves, depicted the absorption characteristics of bare (①), amine-functionalized (③), and NIR-Cu-SiNPs, respectively.

We synthesized NIR-Cu-SiNPs following the procedure outlined in Scheme 1. Initially, silica nanoparticles (1) were generated through the hydrolysis of tetraethyl orthosilicate employing an ammonia solution. Subsequently, the introduction of Cu^2+^ onto the silica nanoparticle surface occurred via chelation between cations and anionic silanol groups (2) [25], followed by amine functionalization with 3-aminopropyl triethoxysilane (3). The final step involved an amide bond formation through an EDC/NHS coupling reaction with the carboxylic acid moiety at the terminal end of the tumor-specific NIR probes, resulting in the formation of NIR-Cu-SiNPs. Scanning electron microscopy (SEM) images illustrated the uniform morphology of NIR-Cu-SiNPs (Figure 1a). Additionally, dynamic light scattering (DLS) measurements yielded a diameter of 163.7 ± 1.04 nm with monodispersity (Figure 1b). Zeta potential analyses reflected the surface modifications: silica nanoparticles exhibited a zeta potential of −43.3 ± 0.74 mV, which increased to −24.0 ± 1.66 mV after amine functionalization. Ultimately, NIR-Cu-SiNPs displayed a zeta potential of −7.58 ± 0.62 mV, reflecting the conversion of amine functional groups to amide bonds with tumor-specific NIR probes. Transmission electron microscopy (TEM) imaging of NIR-Cu-SiNPs, along with energy-dispersive X-ray spectroscopy (EDS) mapping, revealed the presence of Si, O, and Cu elements in a spherical morphology (Figure 1c). The characteristics of intermediate nanoparticles in Scheme 1 are provided in the Supplementary Materials (Table S1 and Figure S1).

We conducted the quantitative analysis of Cy5.5 within the tumor-specific NIR probe incorporated into NIR-Cu-SiNPs using UV-vis spectroscopy (Figure 1d). Silica nanoparticles (1) and amine-functionalized silica nanoparticles containing Cu^2+^ ions (3) did not display characteristic peaks within the 500–800 nm range. Conversely, the absorption curve of NIR-Cu-SiNPs exhibited an absorption peak at 600 nm, corresponding to Cy5.5. To determine the quantity of the tumor-specific NIR probe on silica nanoparticles, we measured the absorbance of unreacted Cy5.5 after its conjugation with amine-functionalized silica nanoparticles and calculated the concentration using the standard curve of the tumor-specific NIR probe. The analysis revealed that each silica nanoparticle harbored 8.67 × 10^−18^ mol of tumor-specific NIR probes.

**Figure 2:**
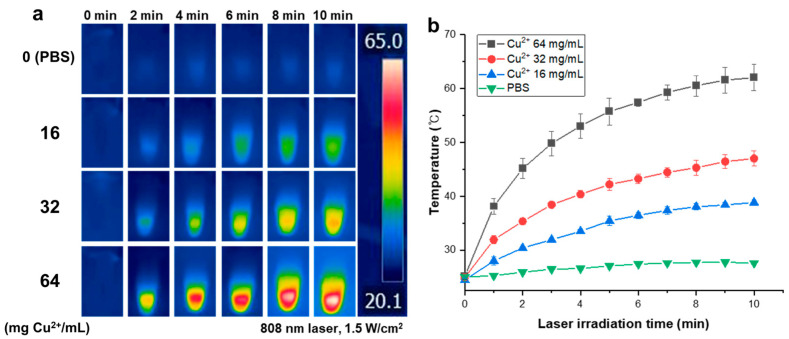
Photothermal properties of NIR-Cu-SiNPs under the irradiation of an 808-nm CW laser at 1.5 W/cm^2^. (**a**) Thermographic images of NIR-Cu-SiNPs depicted the temperature rise corresponding to irradiation time and NIR-Cu-SiNP concentration. (**b**) The temperature increase profile of NIR-Cu-SiNPs during laser irradiation was analyzed.

NIR-Cu-SiNPs exhibited a heat generation response under 808 nm laser irradiation, as illustrated in Figure 2. Even though the absorbance of NIR-Cu-SiNPs was the lowest at 800 mn in Figure 1d, the temperature of NIR-Cu-SiNPs increased up to 62 °C within a 10-min laser irradiation period at a concentration of 64 mg Cu^2+^/mL. This temperature elevation was markedly influenced by the duration of laser irradiation. Furthermore, a decrease in the concentration of Cu^2+^ ions resulted in a slower rate of temperature rise. The observed photothermal effect originated from the d-d transition of Cu^2+^ ions during laser irradiation [26]. Notably, PBS lacking Cu^2+^ ions showed no significant temperature changes. Consequently, this photothermal effect holds promise for cancer treatment, leveraging the vulnerability of cancer cells to heat, which can be modulated by adjusting the irradiation time and NIR-Cu-SiNPs concentration.

**Figure 3:**
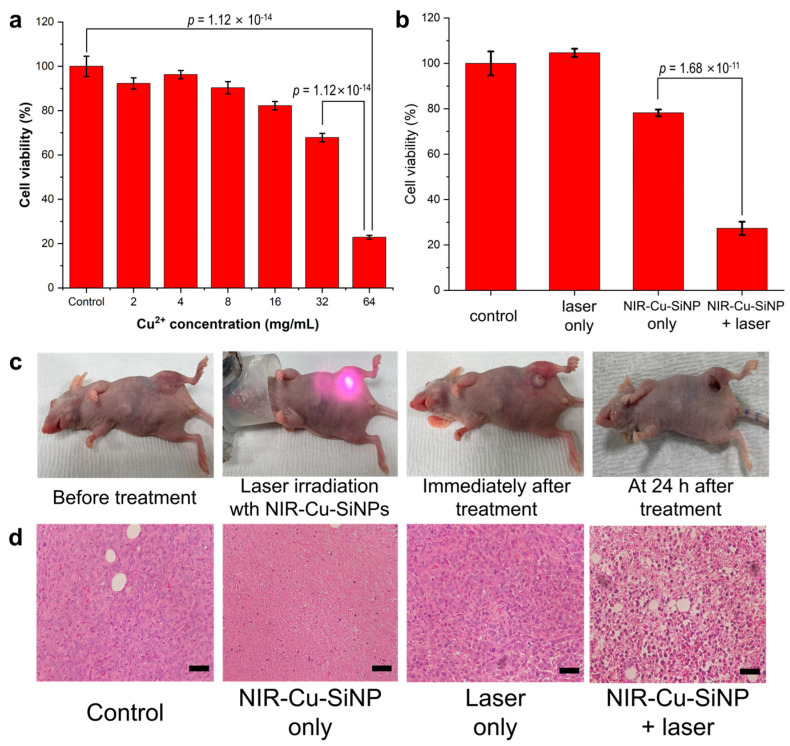
(**a**) Cell viability of 4T1 cells exposed to various concentrations of NIR-Cu-SiNPs. The cellular uptake time of the nanoparticles was 4 h. (**b**) A reduction in cell viability of 4T1 cancer cells under laser irradiation (1.5 W/cm^2^, 10 min) with NIR-Cu-SiNPs, attributable to the photothermal effect. (**c**) In vivo experiment for photothermal therapy on a 4T1-inoculated Balb/c nude mouse. (**d**) Histology analysis of tumor tissues post-treatment (scale bar = 20 μm).

The biocompatibility of NIR-Cu-SiNPs was assessed using 4T1 murine breast cancer cells across various concentrations (Figure 3a). Until reaching a concentration of 32 mg Cu^2+^/mL, NIR-Cu-SiNPs demonstrated minimal cytotoxicity. Considering the photothermal outcomes of the laser irradiation and cytotoxicity, the concentration of NIR-Cu-SiNPs at 32 mg Cu^2+^/mL was selected to investigate the thermal damage inflicted on 4T1 cells. A one-way analysis of variance (ANOVA) was performed to analyze the effect of NIR-Cu-SiNPs on cell viability. The results revealed that there was a statistically significant difference in cell viability by the concentration of NIR-Cu-SiNPs (F(6,28) = 389.20, *p* = 1.26 × 10^−25^). Tukey’s HSD Test for multiple comparisons found that the cell viability of 64 mg Cu^2+^/mL group was significantly lower than other groups (e.g., control vs. 64 mg Cu^2+^/mL, *p* = 1.12 × 10^−14^, 95% C.I. = [70.31, 79.16]; 32 mg Cu^2+^/mL vs. 64 mg Cu^2+^/mL, *p* = 1.12 × 10^−14^, 95% C.I. = [49.29, 58.15]).

Following treatment with NIR-Cu-SiNPs and subsequent irradiation with an 808 nm laser, cancer cells succumbed to damage induced by the photothermal effect of the nanoparticles (Figure 3b and Figure S2, see Supplementary Materials). In contrast, neither NIR-Cu-SiNPs alone nor laser irradiation alone exhibited significant cytotoxicity. A two-way ANOVA was performed to analyze the effect of laser irradiation and NIR-Cu-SiNPs. The results indicated a significant main effect for both NIR-Cu-SiNPs(F(1,12) = 564.20, *p* = 1.86 × 10^−11^) and laser irradiation (F(1,12) = 298.98, *p* = 7.57 × 10^−10^); and a significant interaction between NIR-Cu-SiNPs and laser irradiation (F(1,12) = 396.70, *p* = 1.46 × 10^−10^). Post hoc testing using Tukey’s HSD indicated that cell viability was significantly lower when cells were irradiated by laser after the cellular uptake of NIR-Cu-SiNPs than when they were only irradiated by laser (*p* = 1.63 × 10^−12^) and exposed to only NIR-Cu-SiNPs (*p* = 1.68 × 10^−11^).

Based on the in vitro results of the photothermal effect, we assessed the therapeutic effect on 4T1-bearing balb/c nude mice (Figure 3c). NIR-Cu-SiNPs were injected intratumorally, followed by 10 min exposure to an 808 nm laser. The damage to the tumor tissue was obvious 24 h after the treatment. Then, the tumor tissues from each treated mouse were collected, and the therapeutic effects were evaluated through histological analysis (Figure 3d). The samples treated with PBS (control), only NIR-Cu-SiNPs, or only laser did not exhibit any noticeable damage, while the tissues treated with both NIR-Cu-SiNPs and laser irradiation showed severe tissue damage due to the photothermal effect of NIR-Cu-SiNPs. These results suggested that NIR-Cu-SiNPs possess a good photothermal effect for thermal therapy against cancer.

**Figure 4:**
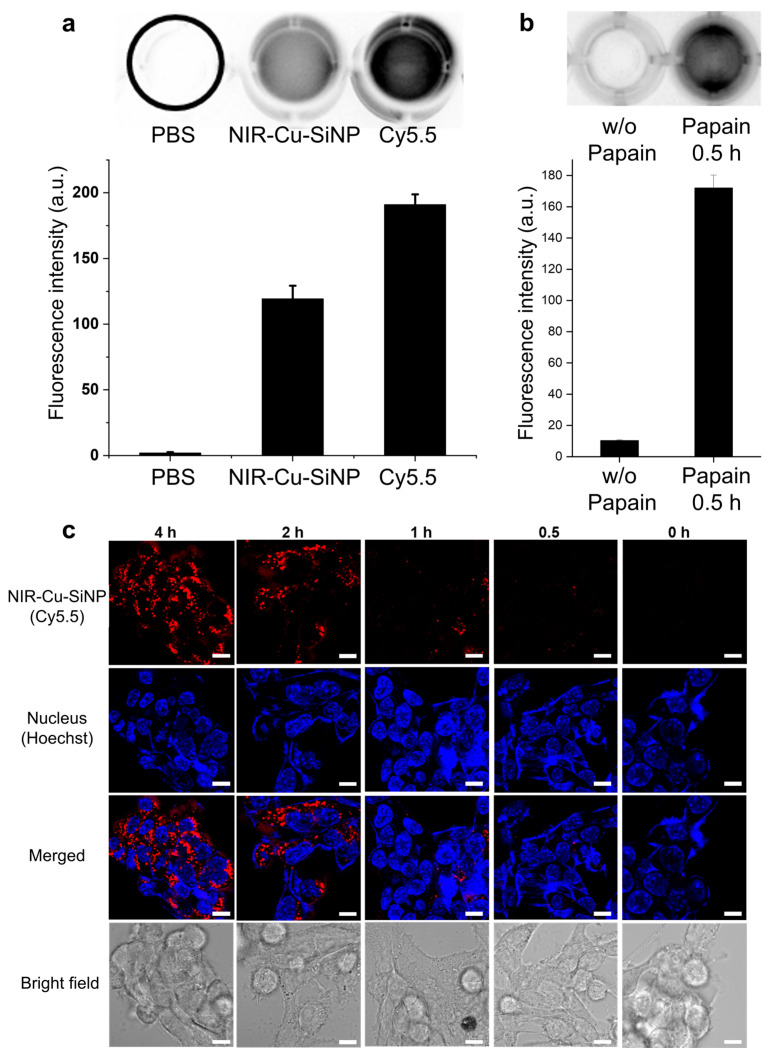
(**a**) Fluorescence images of PBS, NIR-Cu-SiNP, and Cy5.5 along with their corresponding fluorescence intensities. (**b**) Fluorescence images and intensities after the treatment of papain on NIR-Cu-SiNPs for 30 min. (**c**) Confocal microscopic images of 4T1 cells after treatment with NIR-Cu-SiNPs for various incubation times. The red and blue color corresponded to the fluorescence of Cy5.5 and nucleus (Hoechst), respectively (Scale bar = 44 μm).

In addition to the photothermal effect, NIR-Cu-SiNPs also possessed fluorescence quenching properties mediated by Cu^2+^ ions. This quenching phenomenon was harnessed for tumor-specific fluorescent imaging by incorporating the Cy 5.5 fluorescent dye onto the surface of Cu^2+^-loaded silica nanoparticles using a GFLG peptide sequence as a linker. Under normal conditions, the fluorescence of Cy 5.5 was suppressed by Cu^2+^ ions when the fluorophores were in proximity to the nanoparticles (Figure 4a). In comparison to a Cy5.5 solution of equivalent concentration, the fluorescence intensity diminished by approximately 40%. Subsequently, fluorescence recovery was assessed using papain, a cysteine enzyme known for its overexpression in cancer cells [22]. Upon cleaving the GFLG peptides located between Cy5.5 and NIR-Cu-SiNPs, fluorescence rapidly recovered within 30 min (Figure 4b). Conversely, minimal fluorescence was observed in the absence of papain enzymatic activity. The exceptional quenching efficacy of NIR-Cu-SiNPs significantly attenuated background signals, thereby enhancing tumor visibility with improved contrast in fluorescence imaging.

The NIR-Cu-SiNPs exhibited a quenching effect, which was subsequently employed for cancer cell imaging. Following the cellular uptake of NIR-Cu-SiNPs by 4T1 cancer cells, time-dependent fluorescence recovery was observed using confocal microscopy (Figure 4c). Initially, fluorescence was quenched up to 1 h post-cellular uptake, then increased its intensity during 4 h of incubation. Cy5.5 fluorescence was localized in the cytoplasm without overlap with the nucleus. This phenomenon was attributed to the decomposition of the GFLG peptide linker by cysteine enzymes within the cytoplasm, resulting in the release of Cy5.5 molecules and the restoration of their intrinsic fluorescence. Notably, the fluorescence recovery mediated by cysteine enzymes was significantly faster in cancer cells compared to normal cells owing to the overexpression of these enzymes in cancer cells. In contrast, fluorescence imaging of HDF revealed minimal Cy5.5 fluorescence even at 4 h post-cellular uptake (Figure S3, see Supplementary Materials).

**Figure 5:**
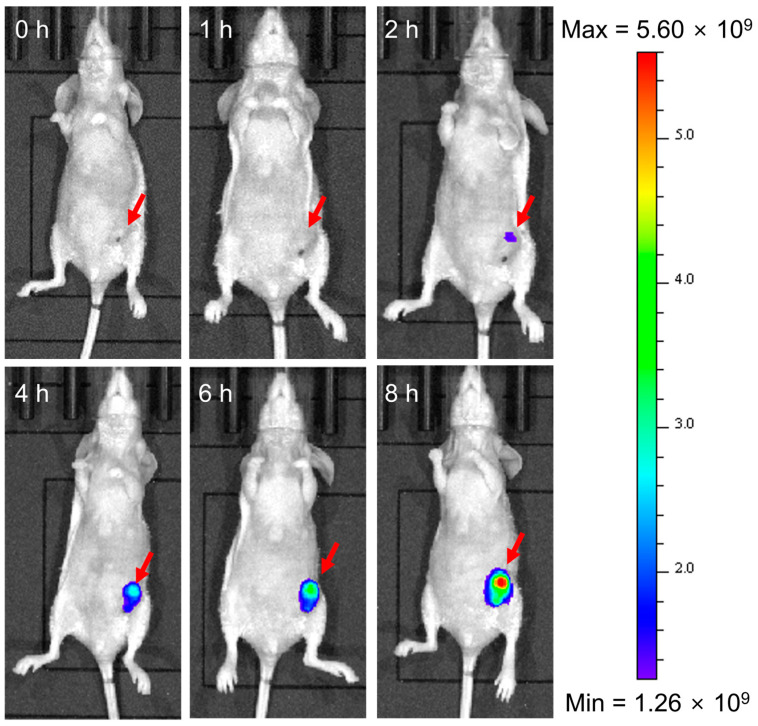
IVIS imaging of 4T1-bearing mice treated with intratumoral injections of NIR-Cu-SiNPs. The red arrow indicates the location of the injection site.

We assessed tumor-specific cancer imaging by intratumoral administration of NIR-Cu-SiNPs to mice with inoculated 4T1 cancer cells (Figure 5). Similar to the in vitro experiment, the in vivo experiment demonstrated fluorescence quenching until 2 h post-injection. Subsequently, the tumor exhibited fluorescence from NIR-Cu-SiNPs, with intensity increasing until 8 h after injection, allowing for the delineation of the tumor. While fluorescence recovery was observed within 1 h in the in vitro experiment, the tumor tissue treated with NIR-Cu-SiNPs began emitting signals 2 h after injection. We attribute this differing temporal behavior of NIR-Cu-SiNPs to variations in the cancer microenvironment between in vitro and in vivo experimental conditions.

## 4. Conclusions

We devised a synthesis process to produce silica nanoparticles incorporating Cu^2+^ ions and conducted a comprehensive analysis of their characteristics. The introduction of Cu^2+^ ions onto uniformly sized silica nanoparticles occurred without inducing the severe toxicity commonly associated with copper. The Cu^2+^ ions played a pivotal role in both the photothermal effect and fluorescence quenching, as corroborated by in vitro and in vivo experiments. These distinctive properties manifested in the infliction of damage to cancer cells or tumor tissues upon laser irradiation and facilitated tumor-specific fluorescence imaging following degradation by cysteine enzymes. 

Subsequent investigations will concentrate on optimizing the design of Cu^2+^-loaded silica nanoparticles. This optimization may encompass refining fabrication techniques, exploring alternative coatings, and adjusting the ratio of Cu^2+^ ions to enhance photothermal efficiency and biocompatibility. Also, establishing a photothermal therapy method is crucial. This therapeutic method should ensure the optimal balance between effectively targeting cancer cells and minimizing adverse effects on normal tissues. Future research endeavors should focus on the establishment and refinement of such techniques, contributing to the development of safer and more targeted photothermal therapies for cancer treatment. Additionally, it is imperative to examine the long-term toxicity of Cu^2+^-loaded silica nanoparticles for clinical applications. Comprehensive in vitro and in vivo studies are necessary to assess the impact on normal cells, organs, and overall systemic effects. 

As a result, we anticipate that Cu^2+^-loaded silica nanoparticles will demonstrate significant promise as versatile therapeutic and imaging agents for cancer treatment. Exploring synergistic effects by combining Cu^2+^-loaded silica nanoparticles with other therapeutic modalities, such as chemotherapy or immunotherapy, has the potential to enhance the overall efficacy of cancer treatment. Expanding the functionality of Cu^2+^-loaded silica nanoparticles by incorporating additional features, such as drug delivery capabilities or targeting ligands, can further enhance their versatility in cancer therapy. In the realm of biomedical imaging, the integration of Cu^2+^-loaded silica nanoparticles with imaging agents of other modalities, such as iron oxide, iodine, and gold for MRI, CT, or photoacoustic imaging, is a viable avenue. In this manner, our focus is directed towards developing Cu^2+^-loaded silica nanoparticles as a more comprehensive and versatile diagnostic and treatment platform for cancer.

## Data Availability

The data presented in this study are available on request from the corresponding authors.

## Supplementary Materials

The following supporting information can be downloaded at: https://www.mdpi.com/article/10.3390/jfb15040081/s1, Table S1: Size distribution and zeta potentials of nanoparticles during the synthesis of NIR-Cu-SiNPs, Figure S1: SEM images of nanoparticles during the synthesis of NIR-Cu-SiNPs (a) silica nanoparticles ①, (b) ②, (c) ③, and (d) NIR-Cu-SiNPs (scale bar = 500 nm), Figure S2: Live cell imaging of 4T1 cancer cells (1 × 10^4^ cells/well) staining with crystal violet solution after PTT. Each well was treated with 32 mg Cu^2+^/mL of NIR-Cu-SiNPs for 4 h or irradiated an 808-nmlaser (1.5 W/cm^2^) for 10 min, Figure S3: Confocal microscopic images of HDF (human dermal fibroblast) after treatment with NIR-Cu-SiNPs for various incubation times. The red and blue color corresponded to the fluorescence of Cy5.5 (NIR-Cu-SiNP) and nucleus (Hoechst), respectively (Scale bar = 44 μm).
