# Supplementary material for: Applications of Cu2+-Loaded Silica Nanoparticles to Photothermal Therapy and Tumor-Specific Fluorescence Imaging
Source: J Funct Biomater. 2024 Mar 25;15(4):81. doi: 10.3390/jfb15040081 (PMC11051373; doi:10.3390/jfb15040081)
Supplement: Supplementary file 1 [file jfb-15-00081-s001.zip › jfb-2900892-supplementary.pdf]

Journal of Functional Biomaterials

Supplementary Materials

Applications of Cu^{2+}-Loaded Silica Nanoparticles to Photothermal Therapy and Tumor-Specific Fluorescence Imaging

Ji-Ho Park [1,2,†], Yejin Sung [2,†], SeongHoon Jo [3], Seung Ho Lee [1,2], Ju Hee Ryu [2], In-Cheol Sun [2,*] and Cheol-Hee Ahn [1,*]

1 NanoBio Materials Laboratory, Department of Materials Science and Engineering, College of Engineering, Seoul National University, Seoul 08826, Republic of Korea; jikl1028@kist.re.kr (J.-H.P.)
2 Medicinal Materials Research Center, Biomedical Research Division, Korea Institute of Science and Technology, Seoul 02792, Republic of Korea; dpwls319@kist.re.kr (Y.S.)
3 Biomaterials Research Center, Biomedical Research Division, Korea Institute of Science and Technology, Seoul 02792, Republic of Korea
* Correspondence: pfesun@kist.re.kr (I.-C.S.); chahn@snu.ac.kr (C.-H.A.)
† These authors contributed equally to this work.

Table S1. Size distribution and zeta potentials of nanoparticles during the synthesis of NIR-Cu-SiNPs.

Nanoparticles	Mean particle diameter (nm)	Zeta potential (mV)
Silica nanoparticle ①	120.9 ± 0.60	-43.3 ± 0.74
②	162.3 ± 2.81	-32.0 ± 1.52
③	144.6 ± 1.58	-24.0 ± 1.66
NIR-Cu-SiNP	163.7 ± 1.04	-7.58 ± 0.62

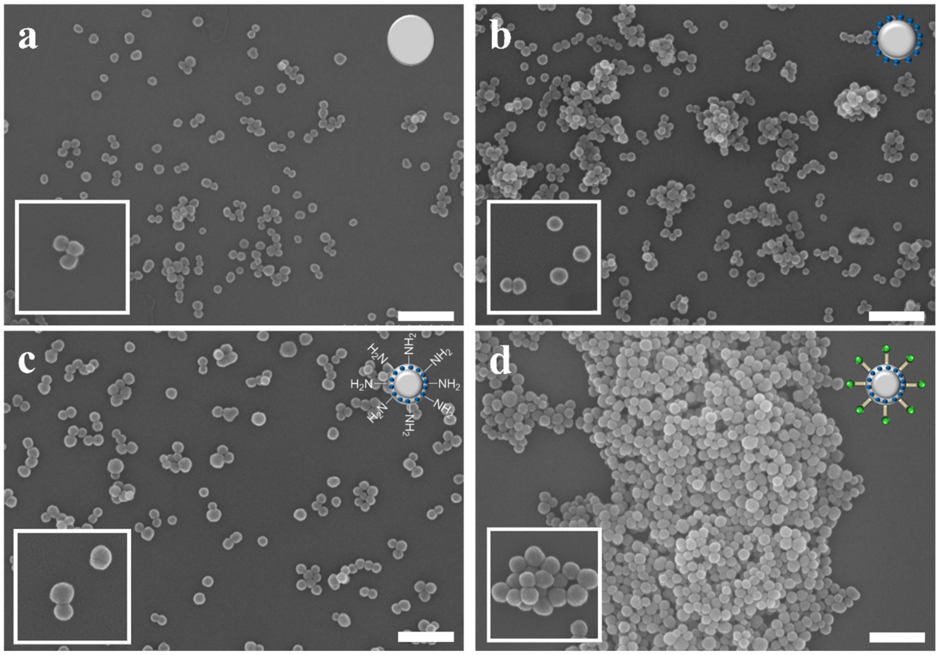

Figure S1. SEM images of nanoparticles during the synthesis of NIR-Cu-SiNPs (**a**) silica nanoparticles ①, (**b**) ②, (**c**) ③, and (**d**) NIR-Cu-SiNPs (scale bar = 500 nm).

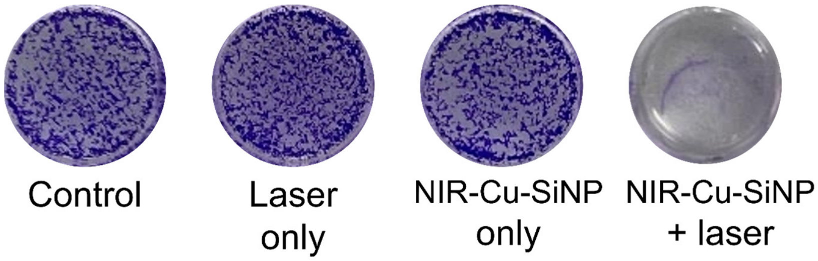

Figure S2. Live cell imaging of 4T1 cancer cells (1x10^4 cells/well) staining with crystal violet solution after PTT. Each well was treated with 32 mg Cu^{2+}/mL of NIR-Cu-SiNPs for 4 h or irradiated an 808-nm laser (1.5 W/cm^2) for 10 min.

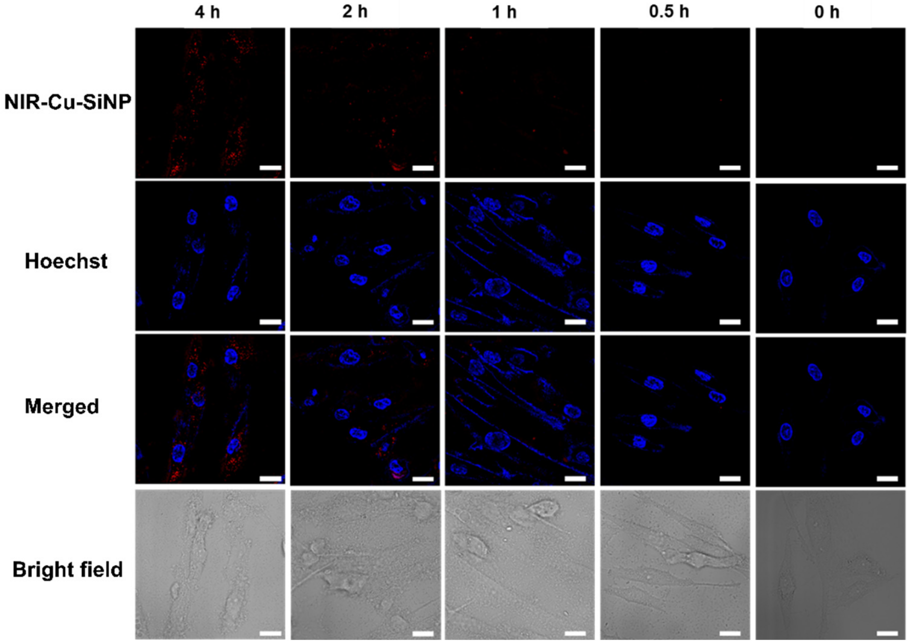

Figure S3. Confocal microscopic images of HDF (human dermal fibroblast) after treatment with NIR-Cu-SiNPs for various incubation times. The red and blue color corresponded to the fluorescence of Cy5.5 (NIR-Cu-SiNP) and nucleus (Hoechst), respectively (Scale bar = 44 μm).
